# Y-27632 targeting ROCK1&2 modulates cell growth, fibrosis and epithelial-mesenchymal transition in hyperplastic prostate by inhibiting β-catenin pathway

**DOI:** 10.1186/s43556-024-00216-9

**Published:** 2024-10-26

**Authors:** Shidong Shan, Min Su, Hejin Wang, Feng Guo, Yan Li, Yongying Zhou, Huan Liu, Lu Du, Junchao Zhang, Jizhang Qiu, Michael E. DiSanto, Yuming Guo, Xinhua Zhang

**Affiliations:** 1 Department of Urology, Zhongnan Hospital of Wuhan University, Wuhan, China; 2 Department of Renal Transplatation, Guangdong Provincial People’ Hospital (Guangdong Academy of Medical Sciences), Southern Medical University, Guangzhou, China; 3 Department of Gynecological Oncology, Zhongnan Hospital of Wuhan University, Wuhan, China; 4 Department of Surgery and Biomedical Sciences, Cooper Medical School of Rowan University, Camden, NJ USA

**Keywords:** Y-27632, Benign prostatic hyperplasia, Cell growth, Fibrosis, Epithelial-mesenchymal transition

## Abstract

Benign prostatic hyperplasia (BPH) is a prevalent condition affecting the male urinary system, with its molecular mechanisms of pathogenesis remaining unclear. Y-27632, a non-isoform-selective Rho kinase inhibitor, has shown therapeutic potential in various diseases but its effects on static factors and fibrosis in BPH remain unexplored. This study investigated human prostate tissues, human prostate cell lines, and BPH rat model using immunofluorescence, flow cytometry, quantitative reverse transcription polymerase chain reaction, western blotting, and cell counting kit-8. ROCK1 and ROCK2 were significantly up-regulated in BPH tissues, correlating with clinical parameters. Y-27632 targeted the inhibition of ROCK1 & ROCK2 expression and inhibited cell proliferation, fibrosis, epithelial-mesenchymal transition (EMT), while induced cell apoptosis in a dose-dependent manner. Moreover, knockdown of either ROCK isoform inhibited fibrosis and EMT, induced apoptosis, while ROCK overexpression had the opposite effects. ROCK downregulation inhibited the β-catenin signaling pathway (such as C-MYC, Snail and Survivin) and decreased β-catenin protein stability, while inhibiting TGF-β/Smad_2/3_ signaling. At the in vivo level, Y-27632 reversed prostatic hyperplasia and fibrosis in BPH model rats to some extent. Our study sheds light on the therapeutic potential of Y-27632 in regulating prostate cell growth, fibrosis and EMT, and demonstrates for the first time the regulatory effect of ROCK isoforms on prostate cells, providing the basis for future research of ROCK isoform-selective inhibitors.

**Supplementary Information:**

The online version contains supplementary material available at 10.1186/s43556-024-00216-9.

## Introduction

Benign prostatic hyperplasia (BPH) is a common condition marked by non-malignant enlargement of human prostate, predominantly affecting aging men and resulting in lower urinary tract symptoms (LUTS), such as hesitancy, weak urine stream, and incomplete emptying in urination. Chronic BPH may lead to bladder dysfunction and subsequent complications, such as urinary retention, recurring urinary tract infections, and ultimately, renal impairment. The prevalence of BPH increases with age, with histological evidence presents in approximately 50% to 60% of men aged 60 and 80% to 90% of men aged 70 and older. The pathophysiology of BPH is multifactorial, involving alterations of estrogen and androgen levels, imbalance of cell growth, inflammation, fibrosis, and EMT [1–3]. Histologically, BPH is marked by an abnormal accumulation of stromal and epithelial cells in the prostate's periurethral area, stemming from dysregulation of cell proliferation and apoptosis. Current BPH treatments, primarily 5α-reductase inhibitors and α-adrenergic receptor antagonists, fail to halt disease progression in approximately 30% of patients, who may eventually require surgical intervention [4, 5]. Thus, exploring new molecular pathologies and alternative therapeutic strategies is crucial, especially for refractory cases.

Rho-associated protein kinase (ROCK), the downstream effector of the small GTPase RhoA, plays a multifaceted role in basic cell functions with two isoforms, ROCK1 and ROCK2, exhibiting distinct functions and tissue distributions [6]. GTP-bound Rho GTPases act on the Rho-binding domain (RBD) of ROCK to cause conformational changes that subsequently activate the kinase [7, 8]. Y-27632, as a non-isoform-selective ATP-competitive Rho kinase inhibitor, has been widely studied for its biological effects [9]. ROCK regulates actin cytoskeleton dynamics through phosphorylation of myosin light chain (MLC). This results in actomyosin contractility, which is essential for cell migration, cytokinesis, and cell proliferation [10, 11]. It is associated with cardiovascular diseases and fibrosis across various pathological conditions [12–16]. In prostate disease research, Y-27632 or ROCK knockdown resulted in a decrease in the motility and proliferation of PC3 prostate cancer cells, while ROCK was linked to the stability of C-MYC protein [17]. Moreover, the up-regulation of ROCK1 was associated with genetic instability as well as poor clinical prognosis of prostate cancer [18]. These findings indicated that ROCK was involved in proliferation and programmed cell death of prostate cells, potentially contributing to the development of BPH. Previous studies on BPH had predominantly focused on the inhibitory action of Y-27632 on prostate contraction, which was verified in both rat and human tissues [19–21]. Other than the tone of smooth muscle, the effects of ROCK on pathogenesis of BPH required further exploration. Importantly, as Y-27632 non-selectively inhibited both isoforms of ROCK, it might lead to systemic side effects such as hypotension [22], underscoring the necessity for more detailed studies on the molecular functions of single ROCK isoform.

The β-catenin signaling pathway is essential for regulating key cellular processes, including growth and differentiation. Wnt signaling inhibits the degradation complex comprising APC, Axin, and GSK-3β, resulting in the stabilization and nuclear translocation of β-catenin, which modulates gene expression [23]. Research showed that β-catenin pathway mediates the proliferative effects of microcystin-leucine-arginine (MC-LR) in human normal prostate epithelial cell line RWPE-1, contributing to prostatic hyperplasia [24]. Additionally, β-catenin signaling was implicated in BPH promotion induced by di-(2-ethylhexyl) phthalate exposure [25]. Elevated level of β-catenin expression was observed in BPH tissues, underscoring its association with prostatic hyperplasia [26]. Furthermore, ROCK-mediated β-catenin accumulation has been reported [27]. TGF-β signaling leads to the phosphorylation of Smad2 as well as Smad3, which were translocated into the nucleus to regulate gene transcription [28]. TGF-β plays an important role in development of BPH by facilitating the differentiation of fibroblasts into myofibroblasts and contributing to the remodeling of stromal cells [29]. Additionally, Immunohistochemical study indicated that TGF-β expression is obviously elevated in BPH tissue compared to normal prostate tissue, primarily sourced from stromal cells [30]. Our study investigated changes in β-catenin and TGF-β pathways to explore their roles in the regulatory network mediated by ROCK.

Our original data showed that ROCK expression level was up-regulated in BPH tissues, Y-27632 regulated the static factors of BPH pathogenesis (including cell proliferation, apoptosis, and EMT) and fibrosis both in vivo and in vitro. For the first time, we found that both ROCK1 and ROCK2 isoforms regulated BPH via β-catenin and TGF-β pathway networks. Our study highlighted Y-27632's therapeutic potential for BPH and provided a theoretical basis for future development of isoform-selective ROCK inhibitors with fewer side effects.

## Results

### ROCK1 and ROCK2 were up-regulated in BPH tissues compared with normal tissues, showing correlations with some clinical parameters

**Fig. 1:**
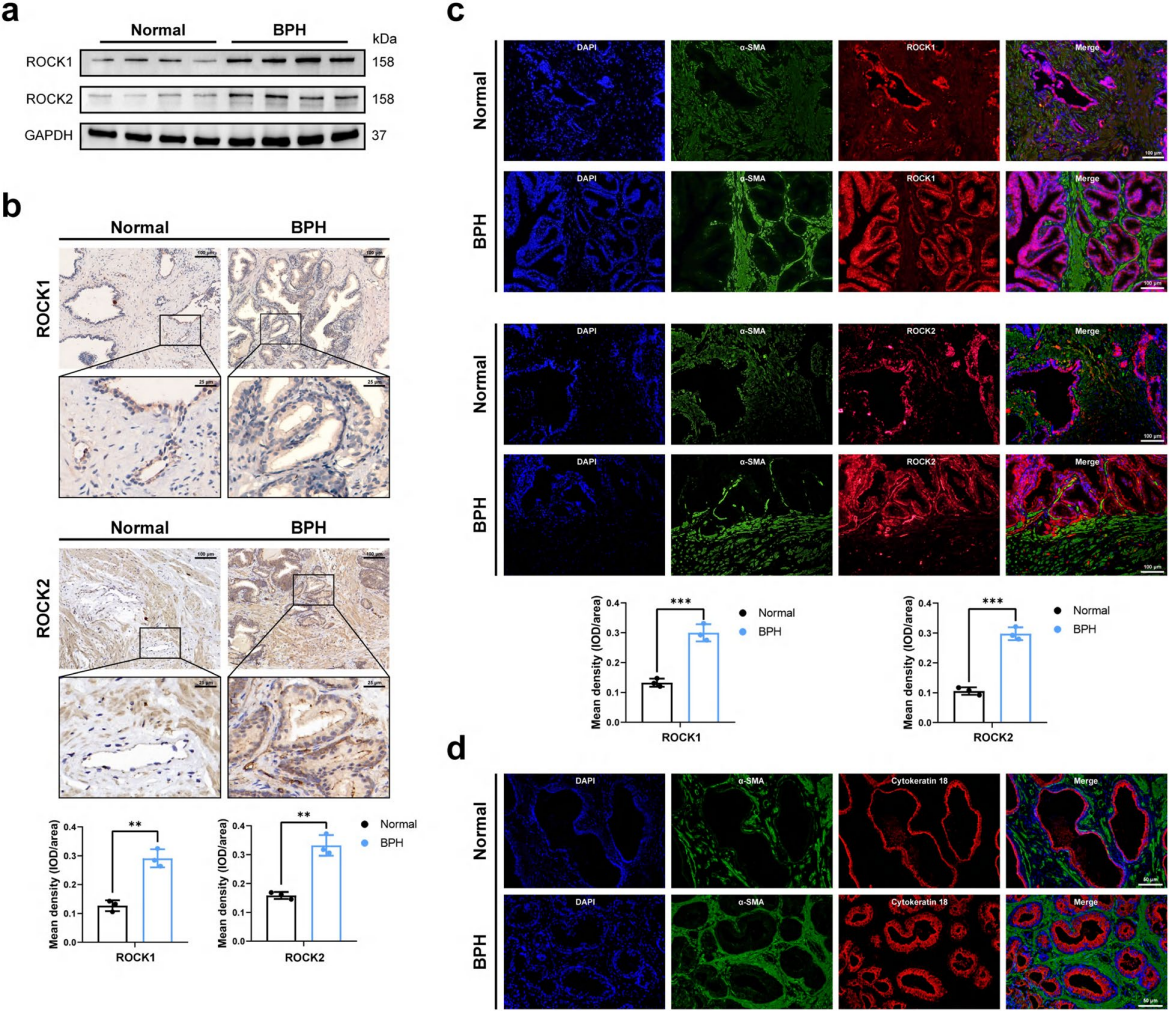
The expression of ROCK1 and ROCK2 in human BPH tissues was higher than that in normal human prostate tissues. **a** Immunoblot assay revealed the protein expression level of ROCK1 and ROCK2 in normal prostate tissue and BPH tissue. **b** Immunohistochemical staining of ROCK1 and ROCK2 for normal human prostate and BPH prostate, the scale bars are 100 μm (upper) and 25 μm (bottom), respectively. (*n* = 3). **c** Immunofluorescence staining of ROCK1 and ROCK2 for normal human prostate and BPH prostate. DAPI (blue) indicated nuclear staining, Cy3-immunofluorescence (red) indicated ROCK1 or ROCK2 protein staining and FITC-immunofluorescence (green) indicated α-SMA protein staining, the scale bars are 100 μm. (*n* = 3). **d** Immunofluorescence staining of α-SMA and Cytokeratin18 for normal human prostate and BPH prostate, α-SMA (green) was used to visualize the stroma and Cytokeratin18 (red) was used to visualize the epithelium, the scale bars are 50 μm. Data were expressed as mean ± SD. ** *p* < 0.01, *** *p* < 0.001

To clarify the interrelation between Rho kinase and human BPH, we detected its expression and localization. Western blotting indicated that both ROCK1 and ROCK2 proteins expression were increased in BPH tissues than that in normal prostate tissues (Fig. 1a). Immunohistochemistry and immunofluorescence staining showed that ROCK1 and ROCK2 were located in both stromal and epithelial components of human prostate, and both were up-regulated in BPH tissues (Fig. 1b, c). To help better distinguish the epithelium from the stroma, we supplemented immunofluorescence co-staining, to determine the epithelial region of the human prostate by cytokeratin18 and the stromal region by α-SMA (Fig. 1d). We then quantified the percentage of positive areas for ROCK1 and ROCK2 proteins on tissue microarray (TMA), and assessed the correlation between their expressions and clinical parameters (Fig. S1). There was a positive correlation between ROCK1 and total PSA (tPSA) (Prostate-Specific Antigen, PSA), and a negative correlation with the free-to-total PSA ratio (fPSA/tPSA). ROCK2 exhibited positive correlations with both fPSA and tPSA, but no significant association with the fPSA/tPSA ratio. PSA levels in patients with BPH are generally higher than normal (because BPH causes the prostate to increase in volume, more prostate tissue secretes more PSA), but lower than those found in most prostate cancer patients. Interestingly, a positive correlation was observed between ROCK2 expression levels and patient age. The Masson staining in TMA showed that the degree of fibrosis was positively correlated with age and not significantly correlated with other clinical parameters (Fig. S2). Furthermore, immunofluorescence staining and TUNEL staining of human prostate tissues showed that N-Cad and Collagen I were up-regulated, while E-Cad was down-regulated in BPH tissues. Moreover, the positive rate of TUNEL was also down-regulated in BPH tissues (Fig. S3). These results suggested that ROCK1 and ROCK2 were up-regulated in BPH, expressed in both stroma and epithelium, and the degree of fibrosis and EMT was increased while the degree of apoptosis was decreased in BPH tissues.

### Rho kinase inhibitor Y-27632 treatment targeted ROCK1 and ROCK2, and inhibited the proliferation of human prostate cells.

**Fig. 2:**
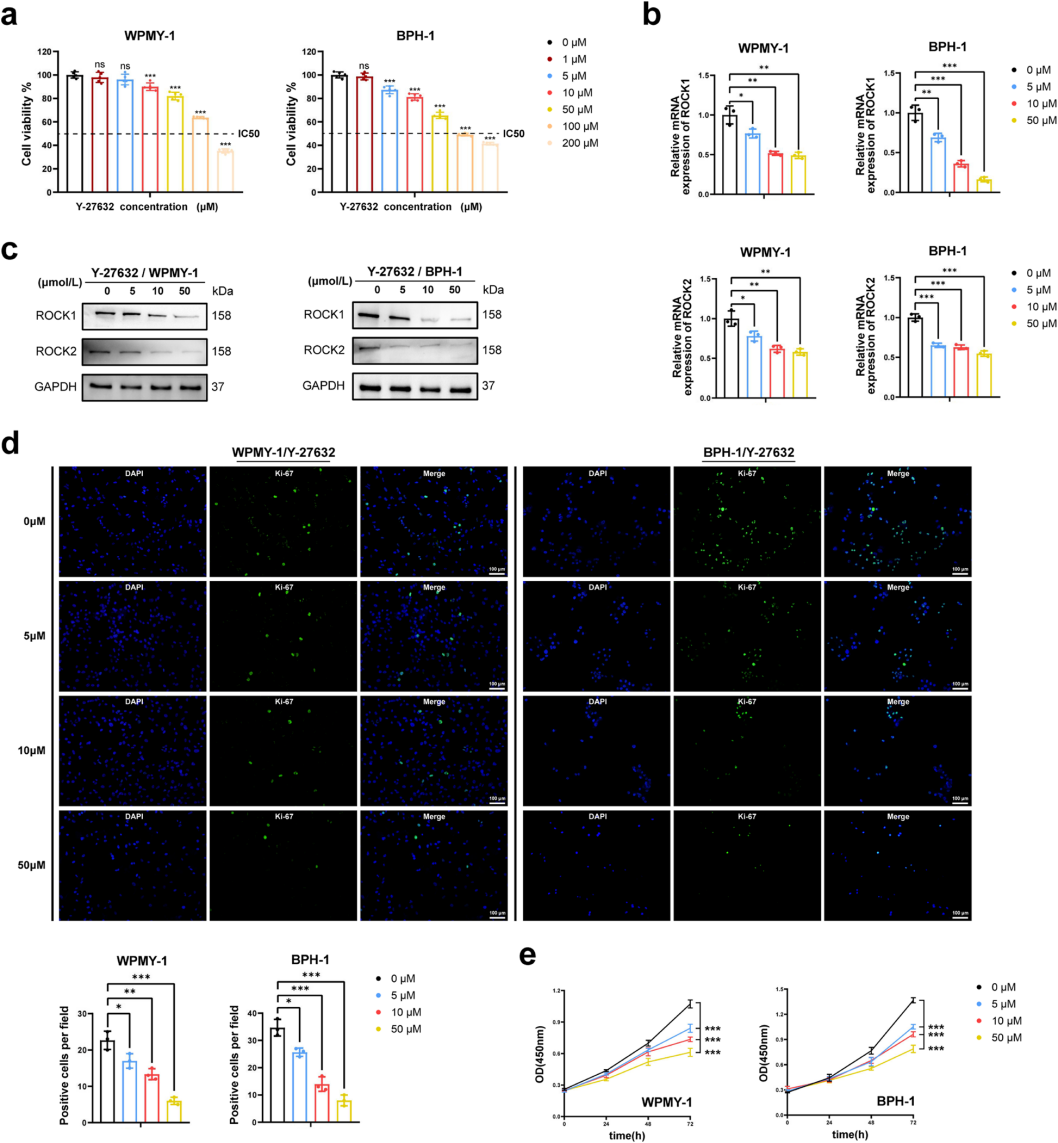
Human prostate cells with Y-27632 treatment inhibited cell proliferation. **a** The inhibition of proliferation by Y-27632 from 0 to 200 μM and the IC50 for the cytotoxic effect of WPMY-1 and BPH-1. (*n* = 5). **b** The relative mRNA level of ROCK1 and ROCK2 in WPMY-1 and BPH-1 after Y-27632 treated for 48 h. (*n* = 3). **c** The relative protein level of ROCK1 and ROCK2 in WPMY-1 and BPH-1 after Y-27632 treated for 48 h. **d** The Ki-67 staining of WPMY-1 and BPH-1 after Y-27632 treated for 48 h. (*n* = 3). **e** The cell viability of WPMY-1 and BPH-1 after Y-27632 treated at different time points by CCK-8 assay. Data were expressed as mean ± SD. ns means no significant difference, * *p* < 0.05, ** *p* < 0.01, *** p < 0.001. The scale bars are 100 μm

Since ROCK was expressed in both epithelial and stromal of prostate tissue, we used prostate epithelial and stromal cell lines for a subsequent series of in vitro studies. The expression of ROCK1 and ROCK2 in two prostate cell lines (WPMY-1 & BPH-1) was verified by immunofluorescence (Fig. S4). We then treated prostate cells with Y-27632 for 48 h at each concentration (0, 1, 5, 10, 50, 100 and 200 μM). CCK-8 was used to evaluate cell viability following Y-27632 treatment, then half-maximal inhibitory concentration (IC50) of Y-27632 associated with cytotoxic effect was estimated (Fig. 2a). We selected three concentrations (5, 10, and 50 μM) lower than IC50 in two cell lines to help rule out the influence of drug toxicity on cell apoptosis or proliferation at high concentrations. In view of the reported nonselective inhibition of ROCK isoforms by Y-27632, we determined the expression changes of ROCK in response to different concentrations of Y-27632 treatment. Our data illustrated that both ROCK1 and ROCK2 were dose-dependently down-regulated by Y-27632 at mRNA and protein levels in both cell lines (Fig. 2b, c). CCK-8 data indicated that Y-27632 dose-dependently inhibited prostatic cell viability with maximal inhibitory effect reached at 72 h (*p* < 0.001) (Fig. 2e). Additionally, the number of Ki-67-positive cells in each field was reduced in prostate cells with Y-27632 treatment, with inhibitory effect enhanced by the increase of treatment concentration (Fig. 2d). The above results showed that Y-27632 targeted ROCK1 and ROCK2 to inhibit proliferation of both prostate cell lines.

### Y-27632 treatment induced human prostate cell apoptosis while inhibited fibrosis and EMT process

**Fig. 3:**
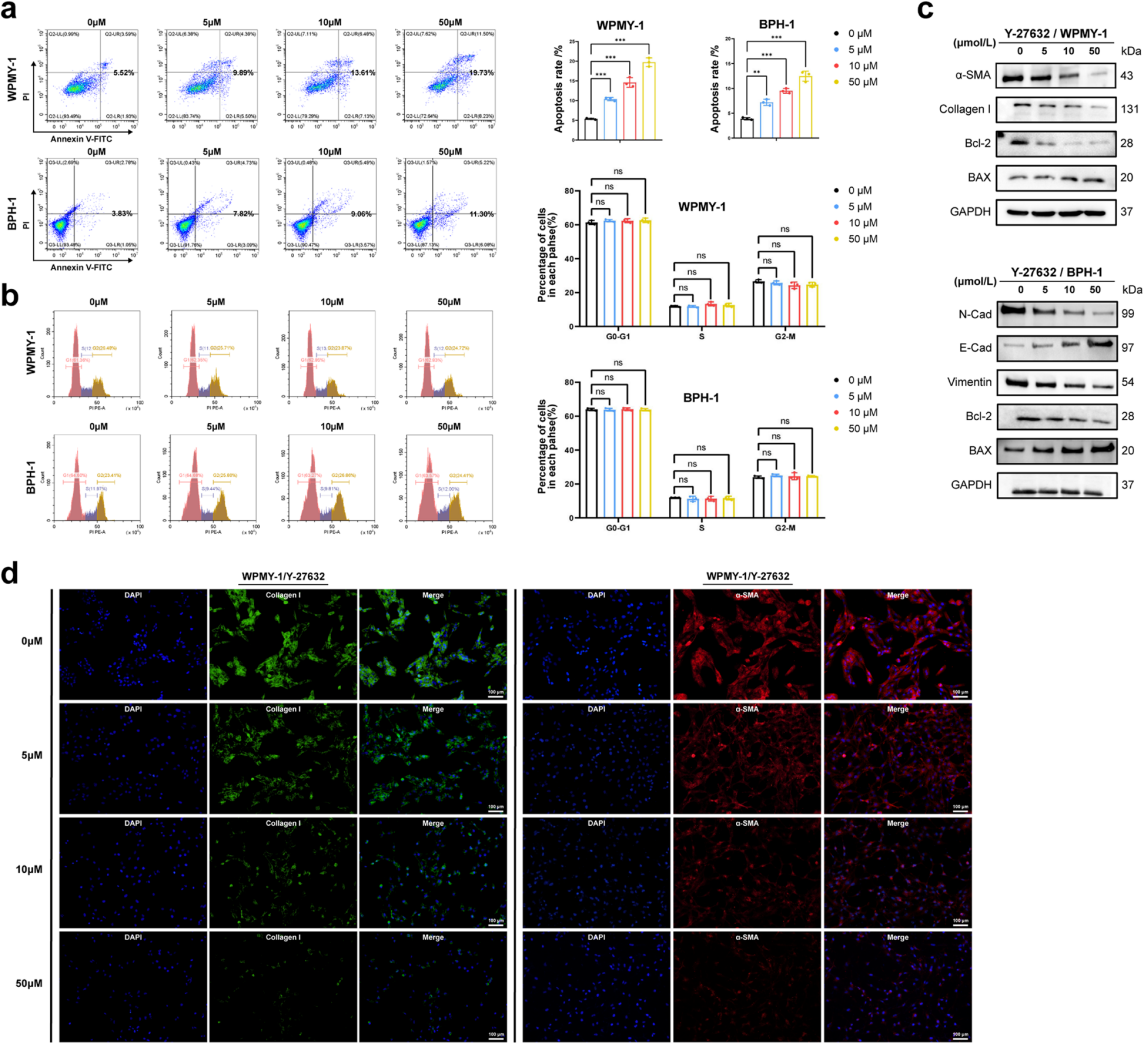
Human prostate cells with Y-27632 treatment induced cell apoptosis, inhibited fibrosis and EMT. **a** Flow cytometry analysis of the cell apoptosis in WPMY-1 and BPH-1 after Y-27632 treated for 48 h. (*n* = 3). **b** Flow cytometry analysis of the cell cycle in WPMY-1 and BPH-1 after Y-27632 treated for 48 h. (*n* = 3). **c** Immunoblot assay of proteins associated with cell fibrosis (α-SMA and collagen I) and cell apoptosis (BAX and Bcl-2) in WPMY-1 after Y-27632 treated for 48 h. Immunoblot assay of proteins associated with EMT (E-Cad, N-Cad and Vimentin) and cell apoptosis in BPH-1 after Y-27632 treated for 48 h. **d** Immunofluorescence staining of α-SMA and collagen I in WPMY-1 after Y-27632 treated for 48 h. DAPI (blue) indicated nuclear staining, Cy3-immunofluorescence (red) indicated α-SMA protein staining and FITC-immunofluorescence (green) indicated Collagen I protein staining. Data were expressed as mean ± SD. ns means no significant difference, ** *p* < 0.01, *** *p* < 0.001. The scale bars are 100 μm

Next, the influence of Y-27632 on cell apoptosis, cell cycle, fibrosis and EMT of prostate cells were studied in vitro. We found that Y-27632 induced a dose-dependent increase in cell apoptosis rate (Fig. 3a), and decreased Bcl-2 while increased BAX (Fig. 3c). Our data showed no statistical difference in the effects of Y-27632 treatment at different concentrations on per phase of cell cycle (G0-G1, S, and G2-M) (Fig. 3b). Fibrosis commonly occurs in stromal cells, while EMT is a pathological process initiated by epithelial cells. In our subsequent experiments, we chose to study fibrosis in the stromal cell (WPMY-1) and EMT in the epithelial cell (BPH-1). We further indicated Y-27632 down-regulated fibrosis markers (Collagen I and α-SMA) dose-dependently in WPMY-1, while inhibited EMT process with Vimentin and N-Cad decreased, along with E-Cad increased in BPH-1 (Fig. 3c). In addition, the immunofluorescence intensity of Collagen I and α-SMA was weaker after Y-27632 treatment, especially at the 10 μM and 50 μM (Fig. 3d). We furthermore found the prostate cells were deformed and atrophic after Y-27632 treatment in the bright field (Fig. S5a), and Y-27632 disrupted the structure of actin cytoskeleton, especially at high concentration (Fig. S5b). These findings suggested that Y-27632 could induce apoptosis and inhibit both fibrosis and EMT in prostate cell lines.

### ROCK1 knockdown inhibited cell proliferation, fibrosis and EMT, induced cell apoptosis of human prostate cells

**Fig. 4:**
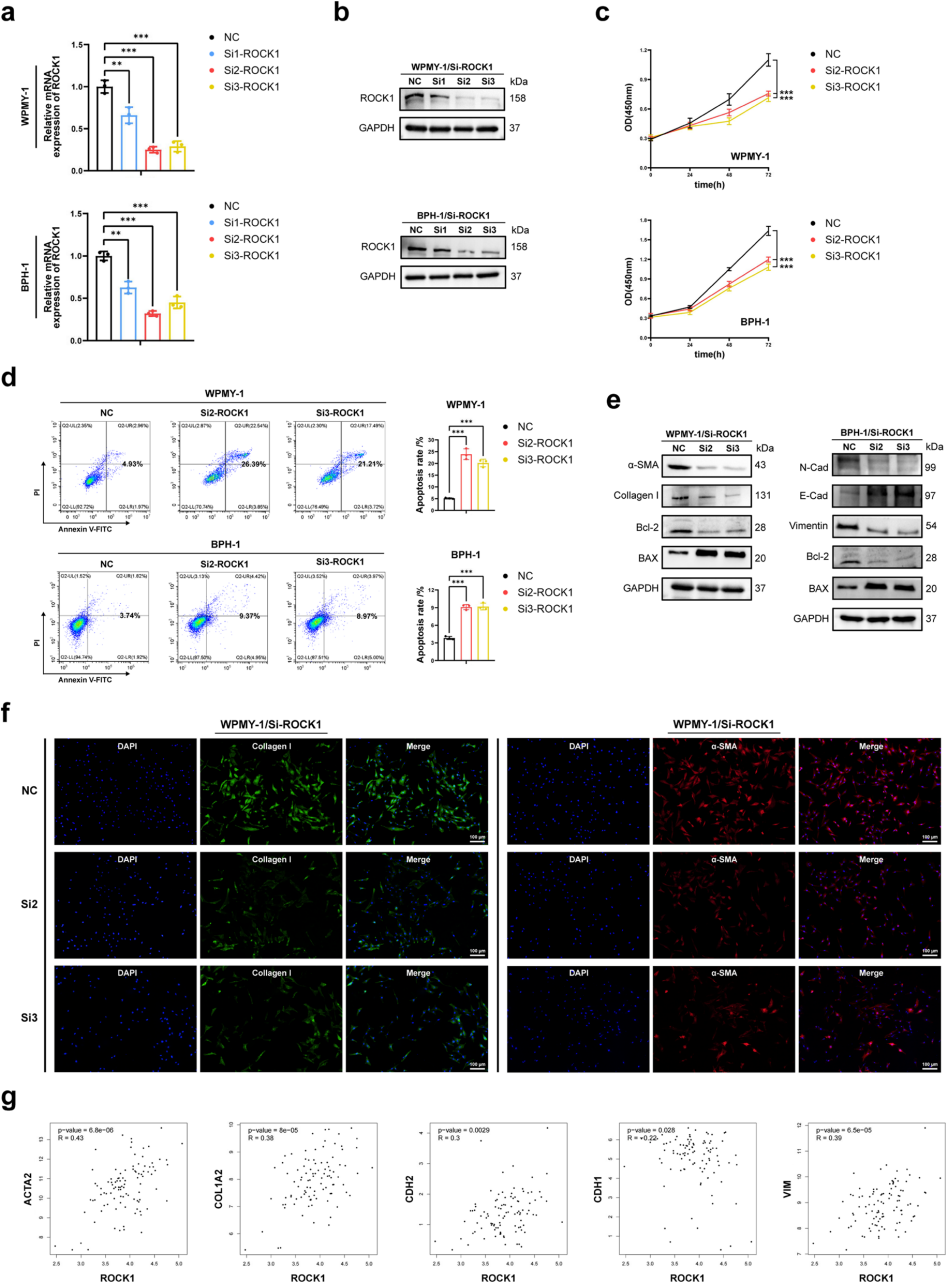
ROCK1 knockdown inhibited cell proliferation, fibrosis and EMT, induced apoptosis of human prostate cells. **a** Knockdown efficiency of ROCK1 at the mRNA level with three different siRNA sequences in WPMY-1 and BPH-1. (*n* = 3). **b** Knockdown efficiency of ROCK1 at the protein level with three different siRNA sequences in WPMY-1 and BPH-1. **c** The cell viability of WPMY-1 and BPH-1 after ROCK1 knockdown at different time points by CCK-8 assay. **d** Flow cytometry analysis of the cell apoptosis in WPMY-1 and BPH-1 after ROCK1 knockdown. (*n* = 3). **e** Immunoblot assay of proteins associated with cell fibrosis and cell apoptosis in WPMY-1 after ROCK1 knockdown. Immunoblot assay of proteins associated with EMT and cell apoptosis in BPH-1 after ROCK1 knockdown. **f** Immunofluorescence staining of α-SMA and collagen I in WPMY-1 after ROCK1 knockdown. DAPI (blue) indicated nuclear staining, Cy3-immunofluorescence (red) indicated α-SMA protein staining and FITC-immunofluorescence (green) indicated Collagen I protein staining. **g** GTEx analysis of the correlation between ROCK1 and ACTA2, COL1A2, CDH2, CDH1 and VIM in prostate. Data were expressed as mean ± SD. ** *p* < 0.01, *** p < 0.001. The scale bars are 100 μm

In an attempt to investigate the function of the single ROCK isoform, we established prostate cell lines with ROCK knockdown using siRNA. qRT-PCR and western blotting were applied to detect the knockdown efficiency at both mRNA and protein levels on prostate cell lines after transfection with three pairs of siROCK1 (Fig. 4a, b). si2-ROCK1 and si3-ROCK1 were selected duo to their high knockdown efficiency. CCK-8 data showed that siROCK1 inhibited viability of both WPMY-1 and BPH-1 with maximal inhibitory effect reached at 72 h (Fig. 4c). Flow cytometry results indicated that siROCK1 obviously increased cell apoptosis rate in prostate cells, with a pronounced pro-apoptotic trend observed in WPMY-1 (Fig. 4d). Consistently, we found that siROCK1 led to the down-regulation of Bcl-2 as well as up-regulation of BAX in prostate cells (Fig. 4e). Western blotting indicated that protein expression level of fibrosis markers in WPMY-1 transfected with siROCK1 was significantly down-regulated (Fig. 4e). Additionally, immunofluorescence images illustrated that siROCK1 alleviated fibrosis in WPMY-1 (Fig. 4f). Furthermore, siROCK1 in BPH-1 decreased the expression of stromal markers, while up-regulated the expression of epithelial marker (Fig. 4e). RNA-seq data (TPM) were obtained from the Genotype Tissue Expression (GTEx) database. After log2 transformation of gene expression levels, Pearson coefficient was calculated. The results demonstrated that in the human prostate, ROCK1 shown different degrees of positive correlation of ACTA2, COL1A2, CDH2 and VIM (α-SMA, Collagen I, N-Cad and Vimentin encoding genes), negatively correlated with CDH1 (E-Cad encoding gene) (Fig. 4g). These data suggested that ROCK1 knockdown promoted apoptosis in prostate cells while inhibiting proliferation, fibrosis, and EMT.

### ROCK2 knockdown, similar to ROCK1 knockdown, inhibited cell proliferation, fibrosis and EMT, induced cell apoptosis of prostate cell lines

**Fig. 5:**
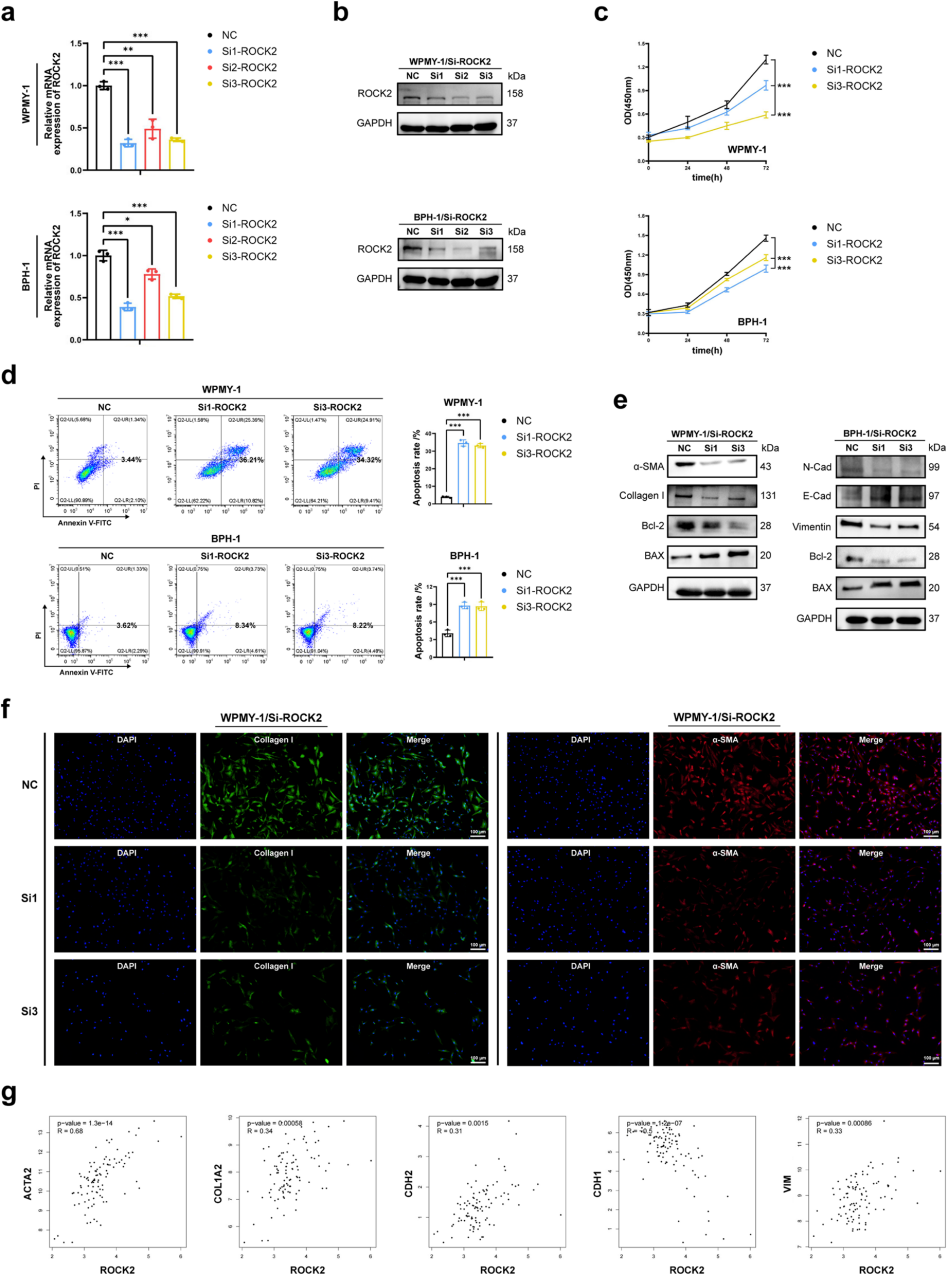
ROCK2 knockdown inhibited cell proliferation, fibrosis and EMT, induced apoptosis of human prostate cells. **a** Knockdown efficiency of ROCK2 at the mRNA level with three different siRNA sequences in WPMY-1 and BPH-1. (*n* = 3). **b** Knockdown efficiency of ROCK2 at the protein level with three different siRNA sequences in WPMY-1 and BPH-1. **c** The cell viability of WPMY-1 and BPH-1 after ROCK2 knockdown at different time points by CCK-8 assay. **d** Flow cytometry analysis of the cell apoptosis in WPMY-1 and BPH-1 after ROCK2 knockdown. (*n* = 3). **e** Immunoblot assay of proteins associated with cell fibrosis and cell apoptosis in WPMY-1 after ROCK2 knockdown. Immunoblot assay of proteins associated with EMT and cell apoptosis in BPH-1 after ROCK2 knockdown. **f** Immunofluorescence staining of α-SMA and collagen I in WPMY-1 after ROCK2 knockdown. DAPI (blue) indicated nuclear staining, Cy3-immunofluorescence (red) indicated α-SMA protein staining and FITC-immunofluorescence (green) indicated Collagen I protein staining. **g** GTEx analysis of the correlation between ROCK2 and ACTA2, COL1A2, CDH2, CDH1 and VIM in prostate. Data were expressed as mean ± SD. * *p* < 0.05, ** p < 0.01, *** *p* < 0.001. The scale bars are 100 μm

Next, we performed functional studies of ROCK2 in prostate cells. qRT-PCR and western blotting were performed to detect the changes in mRNA as well as protein levels in prostate cells following transfection with three pairs of siROCK2 (Fig. 5a, b), si1-ROCK2 and si3-ROCK2 were selected duo to their high knockdown efficiency. CCK-8 data indicated that cell viability transfected with siROCK2 was inhibited, especially at 72 h (Fig. 5c). Flow cytometry analysis showed that siROCK2 induced apoptosis in prostate cells, with a pronounced effect observed in WPMY-1 (Fig. 5d). Consistently, we found that BAX was up-regulated while Bcl-2 was decreased after transfection with siROCK2(Fig. 5e). Western blotting and cellular immunofluorescence staining both showed that the fibrosis markers of WPMY-1 transfected with siROCK2 were significantly down-regulated (Fig. 5e, f). In addition, we found that siROCK2 down-regulated stromal markers, while up-regulated epithelial marker in BPH-1 (Fig. 5e). The data from GTEx suggested that ROCK2 shown different degrees of positive correlation of ACTA2, COL1A2, CDH2 and VIM, negatively correlated with CDH1 (Fig. 5g). The results indicated that ROCK2 knockdown produced effects similar to those observed with ROCK1 down-regulation, including negative regulation of cell proliferation, fibrosis, and EMT, while promoting cell apoptosis.

### β-catenin signaling was involved in the regulation as the downstream of ROCK1 and ROCK2

**Fig. 6:**
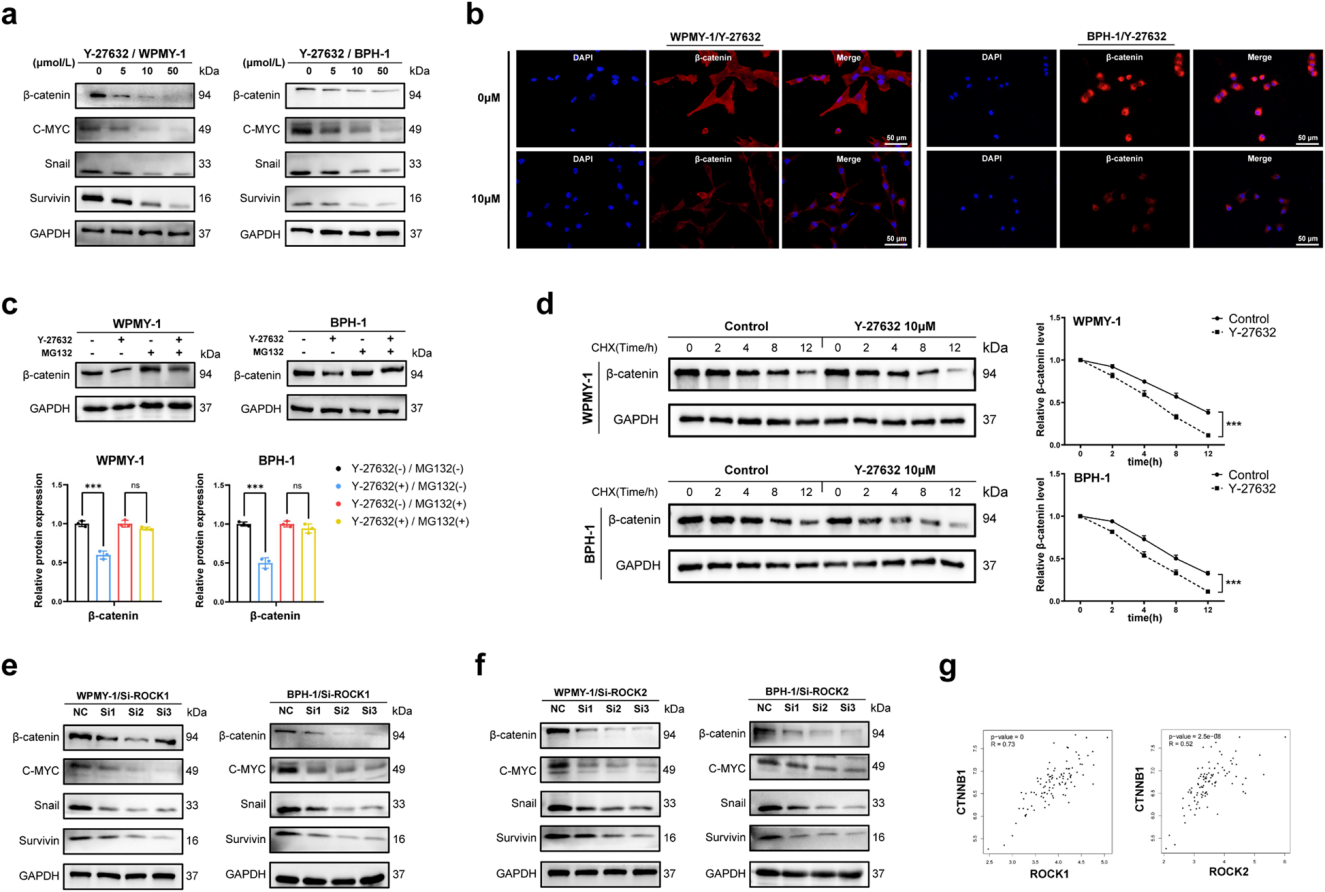
β-catenin signaling axis was involved in the regulation as the downstream of ROCK. **a** Immunoblot assay of proteins associated with the β-catenin signaling axis (β-catenin, C-MYC, Snail and Survivin) in WPMY-1 and BPH-1 after Y-27632 treated for 48 h. **b** Immunofluorescence staining of β-catenin in WPMY-1 and BPH-1 after Y-27632 treated for 48 h. DAPI (blue) indicated nuclear staining and Cy3-immunofluorescence (red) indicated β-catenin protein staining. **c** The expression of β-catenin protein after proteasome inhibition determined by immunoblot assay in WPMY-1 and BPH-1. (*n* = 3). **d**)The stability of β-catenin protein determined by immunoblot assay in WPMY-1 and BPH-1 after Y-27632 treated for 48 h. **e** Immunoblot assay of proteins associated with the β-catenin signaling axis in WPMY-1 and BPH-1 after ROCK1 knockdown. **f** Immunoblot assay of proteins associated with the β-catenin signaling axis in WPMY-1 and BPH-1 after ROCK2 knockdown. **g** GTEx analysis of the correlation between CTNNB1 and ROCK1&2 in prostate. Data were expressed as mean ± SD. ns means no significant difference, *** *p* < 0.001. The scale bars are 50 μm

We further investigated the downstream signaling pathway by which ROCK1 & ROCK2 inhibited cell growth, fibrosis and EMT in prostate cells. Given the critical role of β-catenin signaling in regulating cell growth differentiation and other important basic functions, we first determined the expression of β-catenin in prostate cells following Y-27632 treatment. It was revealed that Y-27632 decreased the β-catenin protein expression level, and the downstream proteins C-MYC, Snail and Survivin were also inhibited, with maximal inhibitory effect reached at 50 μM (Fig. 6a). Cell immunofluorescence images illustrated that 10 μM Y-27632 treatment decreased the fluorescence intensity of β-catenin, especially in nucleus (Fig. 6b). In addition to nuclear translocation, degradation of β-catenin protein was considered another way modulating β-catenin signaling. We use MG132 (a proteasome inhibitor) to treat prostate cells and found it attenuated the inhibitory effect of Y-27632 on β-catenin (Fig. 6c). Next, we found the degradation rate of β-catenin in Y-27632-treated cells was faster than that in the control cells after treatment with CHX (a protein synthesis inhibitor) (Fig. 6d). Additionally, the β-catenin protein and its downstream proteins were down-regulated after siROCK1 or siROCK2 in human prostate cell lines, with maximal inhibitory effect reached at 50 μM (Fig. 6e, f). GTEx data shown different degrees of positive correlation between CTNNB1 expression and that of ROCK1 and ROCK2. (Fig. 6g). The results above suggested that ROCK1 and ROCK2 had positive regulatory effects on β-catenin and its downstream target genes.

### ROCK overexpression promoted fibrosis and EMT, inhibited apoptosis, and TGF-β pathway was involved in the regulation of fibrosis

**Fig. 7:**
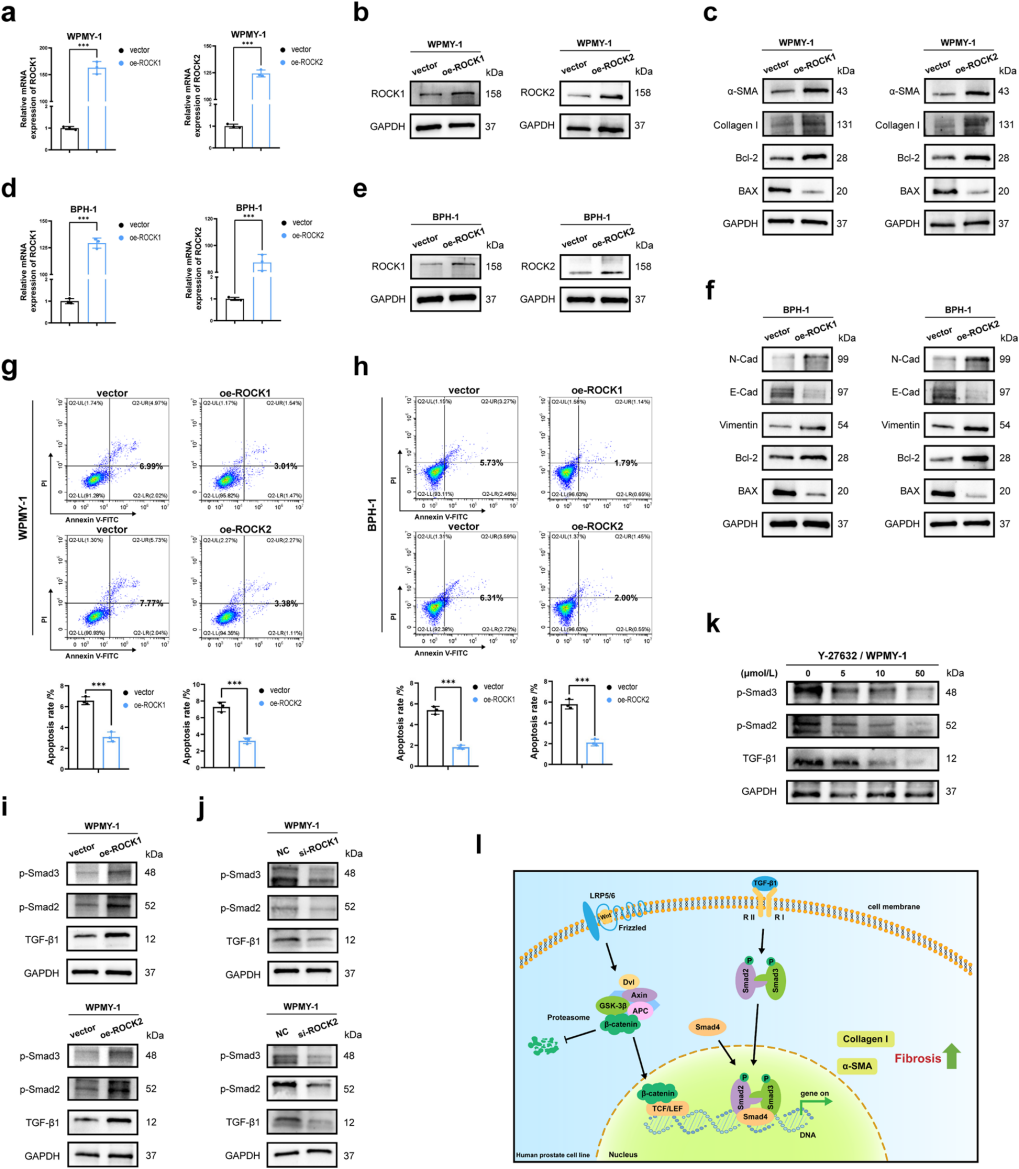
ROCK1 & ROCK2 overexpression promoted fibrosis and EMT, inhibited apoptosis of human prostate cells. TGF-β signaling axis was involved in the regulation of fibrosis.** a** Overexpression efficiency of ROCK at the mRNA level in WPMY-1. (*n* = 3). **b** Overexpression efficiency of ROCK at the protein level in WPMY-1. **c** Immunoblot assay of proteins associated with cell fibrosis and cell apoptosis in WPMY-1 after ROCK overexpression. **d** Overexpression efficiency of ROCK at the mRNA level in BPH-1. (*n* = 3). **e** Overexpression efficiency of ROCK at the protein level in BPH-1. **f** Immunoblot assay of proteins associated with EMT and cell apoptosis in BPH-1 after ROCK overexpression. **g** Flow cytometry analysis of the cell apoptosis in WPMY-1 after ROCK overexpression. (*n* = 3). **h** Flow cytometry analysis of the cell apoptosis in BPH-1 after ROCK overexpression. (*n* = 3). **i** Immunoblot assay of proteins associated with TGF-β signaling (p-Smad3, p-Smad2 and TGF-β1) in WPMY-1 after ROCK overexpression. **j** Immunoblot assay of proteins associated with TGF-β signaling in WPMY-1 after ROCK knockdown. **k** Immunoblot assay of proteins associated with TGF-β signaling in WPMY-1 after Y-27632 treated for 48 h. **l** The simple schematic model proposed for the combined regulation of the β-catenin pathway and the TGF-β pathway on the fibrosis of BPH. Data were expressed as mean ± SD. *** *p* < 0.001

To further validate the impact of ROCK isoforms on prostate cell function, we generated prostate cell lines overexpressing ROCK. First, we confirmed the overexpression efficiency of ROCK1 and ROCK2 plasmids in both cell lines using qRT-PCR (Fig. 7a, d). Subsequently, western blotting analysis confirmed that the overexpression efficiency of ROCK1 and ROCK2 proteins was high (Fig. 7b, e). In WPMY-1, overexpression of ROCK1 or ROCK2 increased the expression of fibrosis markers (Fig. 7c). And in BPH-1, overexpression of ROCK1 or ROCK2 increased N-Cad and Vimentin while decreasing E-Cad (Fig. 7f). Flow cytometry results indicated that overexpression of ROCK1 or ROCK2 reduced apoptosis rates in both cell lines (Fig. 7g, h), consistent with the observed changes in Bcl-2 as well as BAX protein levels (Fig. 7c, f). These results suggested that the effects of ROCK overexpression were opposite to those observed with ROCK knockdown, further confirming the regulatory role of ROCK in human prostate function. Targeting fibrosis during the progression of BPH remains a significant challenge. Therefore, we further investigated the expression of TGF-β signaling pathway. Western blotting showed that overexpression of ROCK1 or ROCK2 in WPMY-1 up-regulated TGF-β1 and its downstream effectors (p-Smad2 and p-Smad3) (Fig. 7i), while knockdown of ROCK1 or ROCK2 showed the opposite trend (Fig. 7j). Consistently, treatment with Y-27632 led to a down-regulation of TGF-β pathway proteins (Fig. 7k). Based on the in vitro results, ROCK might regulate fibrosis progression in BPH through the combined regulation of the β-catenin and TGF-β pathways (Fig. 7l).

### Y-27632 partially reversed the prostatic hyperplasia, fibrosis, proliferation, and apoptosis of BPH rat model

**Fig. 8:**
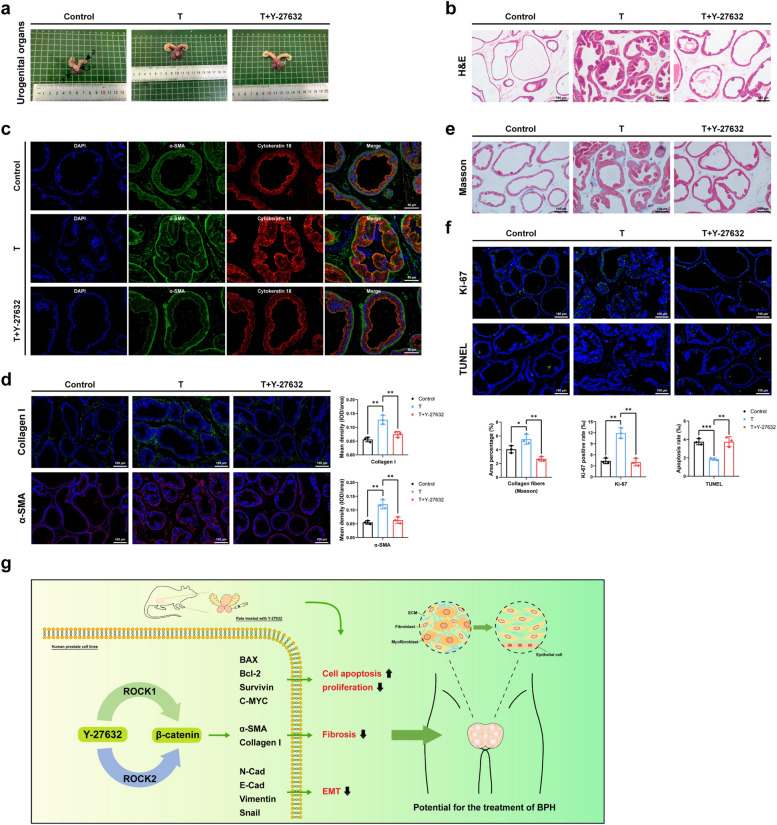
Rho kinase inhibitor Y-27632 partially reversed the prostatic hyperplasia, fibrosis, proliferation, and apoptosis of BPH rat model. **a** The rat urogenital tissues from Control, T, and T + Y 27632 rats. (1) ventral prostate, (2) seminal vesicle, and (3) bladder. **b** Representative H&E staining of Control, T, and T + Y 27632 rat prostates, the scale bars are 100 μm. **c** Immunofluorescence staining of α-SMA and Cytokeratin18 for rat prostates, the scale bars are 50 μm. **d** Representative Immunofluorescence staining of Collagen I and α-SMA for rat prostates, the scale bars are 100 μm. (*n* = 3). **e** Masson’s trichrome staining of rat prostates; prostate epithelial cells were stained orange, SM cells were stained red, and collagen fibers were stained blue, the scale bars are 100 μm. (*n* = 3). **f** The Ki-67 staining and the TUNEL staining for the prostates, the scale bars are 100 μm. (*n* = 3). **g** The simple schematic model proposed for the potential and possible mechanism of ROCK in the regulation of BPH (Y-27632 partially reversed the hyperplasia of the rat prostate in vivo. In human prostate cell lines, Y-27632 regulated a series of target genes via the ROCK1&2-β-catenin signaling axis, thereby promoting apoptosis, inhibiting proliferation, fibrosis, and EMT, demonstrating its potential for BPH treatment). Data were expressed as mean ± SD. * *p* < 0.05, ** *p* < 0.01, *** *p* < 0.001

To validate the therapeutic potential of ROCK at the in vivo level, we established rat model of BPH using testosterone (T) and treated the BPH rats with Y-27632 (Y) by injection into the ventral prostate (Fig. S6a). After the 28th day of sacrifice, we observed a significant enlargement of the prostate in the T group compared to the control (C) group, whereas prostate in the Y group showed partial restoration when compared to the T group (Fig. 8a). Notably, the ventral prostate weight and calculated prostate index of rats in T group were obviously greater compared to those in C group (*p* < 0.001), which could be effectively restored by Y-27632 treatment (*p* < 0.01). And the final body weight of the rats in T group decreased compared with C group, while the seminal vesicle weight increased significantly, and the rats in the Y group failed to recover these changes (Fig. S6b). Next, a significant atrophy of the hyperplastic prostate gland was observed by H&E staining treated with Y-27632 compared with the T group (Fig. 8b). To more clearly differentiate between epithelial and stromal regions, we conducted immunofluorescence co-staining in rat prostate tissue. Cytokeratin 18 was used to identify epithelial regions, while α-SMA marked stromal regions (Fig. 8c). Immunofluorescence images illustrated that Y-27632 restored up-regulated fibrosis markers in the prostate of the T group (Fig. 8d). Consistently, Masson staining indicated that the collagen fibers in the prostate of Y group were decreased compared with T group (Fig. 8e). Additionally, our results indicated that the fluorescence intensity of Ki-67 staining in the prostate of T group was stronger while the fluorescence intensity of TUNEL staining was weaker than that in C group, which were both obviously restored by Y-27632 treatment (Fig. 8f). A schematic model for the mechanism of Rho kinase modulating BPH was provided in the end (Fig. 8g).

## Discussion

BPH is a prevalent urological condition among elderly males, and its molecular regulatory pathways and therapeutic targets still need to be comprehensively investigated. Our in vivo and in vitro studies showed that Y-27632 or selective inhibition of ROCK1 and ROCK2 may exert potential therapeutic effects in BPH by regulating cell growth, fibrosis, and EMT.

We first studied the relationship between ROCK expression and BPH, and found that ROCK1 and ROCK2 were localized in both stroma and epithelium of human prostate tissues, with their expression levels up-regulated in BPH tissues. Previous studies had shown a positive regulation of ROCK on dynamic factors in BPH. Y-27632 regulated the remodeling of detrusor smooth muscle in bladder outlet obstruction models [31]. In the organ bath studies of rat prostate, Y-27632 inhibited contraction induced by electric field stimulation (EFS) and exogenous phenylephrine (PE) [32]. Additionally, the relaxation effect of Y-27632 on cholinergic contraction was also observed in mouse prostate [33]. In this research, we focused on the influence of ROCK on BPH static factors and fibrosis. By immunofluorescence and TUNEL staining, we found that compared with normal tissue, EMT and fibrosis in hyperplastic prostate tissue were enhanced, while apoptosis was reduced. Y-27632 inhibited cell proliferation without affecting apoptosis in smooth muscle cells extracted from human hyperplastic prostate tissue [19], but inhibited cell apoptosis in dissociation-induced mouse prostate stem cell studies [34]. Our findings indicated that Y-27632 inhibited cell viability of human normal prostate stromal cell (WPMY-1) as well as human hyperplastic prostate epithelial cell (BPH-1), while induced cell apoptosis dose-dependently. We speculated that the different effect of Y-27632 on proliferation and apoptosis might be related to specific cell types.

After confirming that Y-27632 negatively regulated cell growth in human prostate cell lines, we further explored its effects on fibrosis and EMT. ROCK had been widely reported to regulate fibrosis in various organs or diseases, including the heart, liver, kidney and endometriosis [14, 35–37]. Our novel data demonstrated the anti-fibrosis effect of Y-27632 by detecting changes in fibrosis marker expression. The association between the actin cytoskeleton and fibrosis had been found in organs such as kidney, heart and liver [38–40]. Current data showed that Y-27632 disrupted the actin cytoskeleton of prostate cells, which was the possible mechanism for inhibiting fibrosis. Y-27632 was found to regulate EMT in lens epithelial cells and rat peritoneal mesothelial cells [41, 42]. Previous research showed that both 5 μM and 10 μM Y-27632 inhibited di-n-butyl phthalate (DBP)-induced EMT in rat renal cells NRK-52E [16]. Y-27632 treatment also led to reorganization of actin meshwork at the edges of cells in MCF-7 (human breast cancer cells), increasing cell rigidity and ultimately inhibiting EMT development [43]. As expected, our findings confirmed that Y-27632 not only disrupted actin cytoskeleton but also reversed EMT in human prostate cell lines, as evidenced by cell phalloidine staining and and the detection in mesenchymal and epithelial markers. This was in line with previous finding that Y-27632 inhibited hyperosmotic stress-induced EMT in renal tubular epithelial cells by attenuating focal adhesions (FAs) and altering the actin cytoskeleton [44]. Additionally, in renal fibrosis studies, damaged nephron tubular epithelial cells accumulate in the interstitium as the condition progresses, adopting a fibroblast-like phenotype and losing their epithelial markers [45, 46]. This suggested that EMT and fibrosis were closely interconnected pathological processes, synergistic inhibition of EMT and fibrosis may provide therapeutic benefits for BPH.

Since the inhibition of Y-27632 on the ROCK isoforms is non-selective, we then explored the isoform-specific effects of ROCK1 and ROCK2 in BPH. Previous study found that ROCK2 inhibited chronic inflammation and fibrosis in cases of chronic graft-versus-host disease [47]. Down-regulation of either ROCK isoform had anti-fibrosis effects in mouse models of pulmonary fibrosis [48]. However, neither ROCK1 nor ROCK2 participated in the progression of fibrosis in the experimental autoimmune myocarditis mouse model study [49]. Another study founded that down-regulation of ROCK1 alleviated perivascular fibrosis in hearts of mice [50]. Consequently, the effects of ROCK isoforms varied across different organs and tissues. In the experimental autoimmune prostatitis rat model, the expressions of RhoA and ROCK1 were up-regulated along with collagen deposition [51]. In addition, microRNA‑122 inhibited the cell viability of prostate cancer cells via down-regulating ROCK2 [52], and ROCK1 or ROCK2 knockdown in DU145 or PC-3 cells inhibited cell proliferation [53]. In the current study, we confirmed that knockdown of ROCK1 or ROCK2 inhibited cell viability, fibrosis and EMT, induced apoptosis on prostate cells. In contrast, overexpression of ROCK1 or ROCK2 produced the opposite effects, reinforcing the strategy of targeting each ROCK isoform individually as a potential treatment for BPH. Due to the highly identical kinase domain between ROCK1 and ROCK2 [7], designing inhibitors that selectively target a single isoform had been challenging. Nevertheless, progress had been made in developing selective inhibitors such as SLx-2119, which inhibited ROCK2 at low concentrations, while the effect on ROCK1 only appeared at high concentrations [54]. Moving forward, further pharmacological studies are warranted to elucidate the functional mechanisms of isoform-specific ROCK inhibitors in the development of human BPH.

Subsequently, we investigated the downstream regulatory networks of ROCK1 and ROCK2. It was found that Y-27632 down-regulated β-catenin dose-dependently in prostate cells. Previous studies had reported a reciprocal association between ROCK and β-catenin signal [27, 55]. Our data indicated that Y-27632 reduced β-catenin level by promoting proteasome degradation and weakening protein stability. The downstream proteins of β-catenin signal were also down-regulated by Y-27632 dose-dependently. C-MYC played an important regulatory role in the proliferation of prostate cells, ROCK1 was found to stabilize and activate the transcriptional activity of C-MYC, and ROCK2 could phosphorylate p300 (Acetyltransferase p300) to enhance its activity [17, 56]. Snail was a key marker of EMT [57], and Survivin was up-regulated in BPH as a crucial marker of apoptosis [58]. The changes of these proteins were consistent with the negative regulation of Y-27632 on cell growth, fibrosis and EMT. Further experiments demonstrated that β-catenin pathway was also inhibited after ROCK1 or ROCK2 knockdown, suggesting that both ROCK isoforms might be the upstream regulators. Supporting these findings, our previous research found that RhoA knockdown down-regulated β-catenin expression, whereas RhoA-ROCK did not change after β-catenin overexpression, reinforcing the notion that RhoA-ROCK may be located upstream of β-catenin [59]. However, deeper studies were needed to investigate the effect of ROCK on the ubiquitination and nuclear translocation of β-catenin.

Given the regulatory potential of ROCK in the challenging area of fibrotic treatment for BPH, we further investigated whether the TGF-β pathway played a role in the process. We observed that inhibition of ROCK down-regulated the expression of TGF-β1/Smad_2/3_ signaling, while activation of ROCK showed the opposite trend. The TGF-β pathway is pivotal in driving fibrosis progression in various organs, including chronic kidney disease, liver fibrosis, and idiopathic pulmonary fibrosis [60–62]. In tumor-related studies, overexpression of TGF-β promoted excessive deposition of EMT and extracellular matrix [63]. Previous research demonstrated that TGF-β can mediated the activation of RhoA [64], and the Rho-dependent activation of JNK signaling contributed to Smad activation [65]. Interestingly, prior studies showed that the effector LEF (Lymphoid enhancer factor) of Wnt/β-catenin pathway may co-occupy the CDH1 promoter with Smad proteins, revealing a molecular mechanism through which the Wnt/β-catenin and TGF-β pathways synergistically regulate EMT [66]. We speculate that these two pathways may synergistically regulate fibrosis in BPH, but further research is needed to identify specific synergistic targets within their interaction.

At last, we further performed in vivo experiments based on the in vitro. Targeted inhibition of ROCK had therapeutic potential in a variety of animal models of urinary diseases. Y-27632 was previously identified to attenuates endothelin-1-induced glomerulopathy in the rats [67]. Another Rho kinase inhibitor hydroxyfasudil was found to ameliorate erectile dysfunction induced by radiation therapy in rats [68]. And fasudil was found to down-regulated inflammatory genes expression in diabetic rats [69]. In the prostate, previous studies primarily focused on the inhibition of the contraction by ROCK inhibitor. Our study revealed that intraprostatic injection of Y-27632 partially reversed the weight of the rat's ventral prostate as well as the prostate index. Immunofluorescence staining and Masson staining revealed a marked remission in fibrosis within the treatment group, while Ki-67 staining and TUNEL staining showed that Y-27632 treatment partially rescued the imbalance between apoptosis and proliferation. Our findings underscored the potential of targeting ROCK to treat BPH. In the testosterone-induced BPH rat model, epithelial hyperplasia predominated, as evidenced by tissue immunofluorescence staining for α-SMA and cytokeratin 18. Looking forward, the development of an animal model characterized by stromal hyperplasia or an animal model with conditional ROCK isoform gene knockout is expected to enhance our understanding of the role of ROCK in BPH.

## Conclusions

As illustrated in Fig. 8g, ROCK inhibitor Y-27632 regulated prostate cell proliferation, apoptosis, fibrosis, and EMT. Both ROCK1 and ROCK2 were involved in BPH regulation, with β-catenin and TGF-β forming a downstream regulatory network. Our study confirmed the potential value of ROCK inhibitor for BPH treatment in vitro and in vivo, while laying a foundation for future research on ROCK isoform selective inhibitors in BPH therapy.

## Materials and methods

### Collection of human prostate tissue samples

Human hyperplastic prostate specimens were obtained from eight men (mean age 72.1 ± 4.8 years) who underwent radical cystectomy for invasive bladder cancer. Two independent clinical pathologists confirmed the presence of BPH but no tumor invasion in these samples. Normal prostate specimens were sourced from eight brain-dead men (mean age 28.5 ± 3.1 years) who were organ donors at the Transplantation Medical Center of Zhongnan Hospital. No hyperplasia was found by pathological examination. Approval for the collection of human tissues was granted by the Hospital Human Investigation Committee, and all patients or their legal representatives signed eligible written informed consent. All research involving human subjects strictly adhered to the requirements outlined in the Declaration of *Helsinki*.

### Immunofluorescence (IF) staining

Prostate specimens from human or rats were fixed with 4% paraformaldehyde (PFA) and paraffin embedded. The section thickness was 5 μm. These sections were placed on a slide with a low temperature thermostat (Leica, GER), and fixed with ice-cold acetone for 10 min after air-dried. After washing in phosphate-buffered saline (PBS), sections were incubated with a PBS solution containing 0.1% BSA and 0.2% TritonX-100 for 2 h. The cells were fixed in a similar way to tissue and then 0.1% TritonX-100 was used to make the membrane permeable. Both tissues and cells were subsequently blocked at normal temperature with the help of goat serum. The slices were placed at 4 °C overnight for primary antibody (Table. S1) incubation, followed by PBS washes. Subsequent incubation with fluorescently labeled secondary antibodies (Table. S2) was performed for 1 h. The nucleus was stained with DAPI. Images were taken with a fluorescence microscope (Olympus, JPN).

### Tissue immunohistochemical (IHC) staining

IHC was conducted on paraffin-embedded, PFA-fixed tissue sections by heat-induced antigen extraction in citrate buffer (pH 6.0, 0.01 M). The sections were treated with 3% hydrogen peroxide to help remove endogenous peroxidase activity. Afterward, goat serum was incubated for 1 h to block nonspecific binding. Then, the slices were placed at 4 °C overnight for appropriate primary antibody (Table. S1) incubation. The slices were applied for 2 h using a biotin-bound anti-rabbit secondary antibody at normal temperature. Following this, the sections were stained using diaminobenzidine tetrahydrochloride (Solarbio, CHN), then counterstained using a H&E staining kit (Beyotime, CHN). All sections were photographed using an inverted microscope.

### Hematoxylin–Eosin (H&E) staining and Masson’s trichrome staining

Paraffin sections of rat ventral prostate samples were stained using H&E and Masson’s trichrome staining (prostate smooth muscle cells were stained red, collagen fibers were stained blue, and epithelial cells were stained orange). Images of the stained sections were captured with a fluorescence microscope.

### TUNEL assay

Paraffin sections of human or rat prostate tissue were processed using a TUNEL Kit (PromoCell, GER) following the instruction’s protocol. The nucleus was stained with DAPI. Images of the sections were captured under fluorescence microscope.

### Culture of cells

Human benign prostatic enlargement epithelial cell line (BPH-1) was obtained from the Procell, CHN. Human normal prostate stromal immortalized cell line (WPMY-1) was acquired from the Stem Cell Bank, CHN. BPH-1 were maintained in RPMI-1640 medium and WPMY-1 were maintained in DMEM medium (Gibco, USA), both added with fetal bovine serum (10%) (FBS, Gibco, USA). The above cells were cultured at 37 °C in humidified incubators containing 5% CO_2_. All cells were identified and verified to be mycoplasma-negative cells during the experiments.

### Transfection of cells

Target-specific small interfering RNA (siRNA) for ROCK1 and ROCK2 was obtained from (Genepharma, CHN) with corresponding sequences provided in Table. S3. Plasmids for ROCK1 and ROCK2 overexpression were synthesized by Miaoling Biology (CHN). WPMY-1 or BPH-1 cell lines were seeded in 6-well plates and transfection operation was performed once they reached 50% confluency. The siRNA or plasmid diluted in serum-free medium was mixed with Lipofectamine 2000 biological reagent (Invitrogen, USA) according to the manufacture’s guide. After 20 min incubation at normal temperature, the mixture was added to the appropriate well. After 6–8 h, cell culture medium was replaced with complete growth medium. Transfection efficiency of mRNA and protein was assessed using qRT-PCR and western blotting.

### Cell Counting Kit-8 (CCK-8) assay

A total of 2000 cells were seeded in each well of the 96-well plates and incubated at 37 °C in the dark. The culture medium was refreshed at 0 h, 24 h, 48 h and 72 h. At each of above time points, 10 μL of CCK-8 reagent (Meilune, CHN) was supplemented to each well. After incubating for 1 h at 37 °C, the absorbance at 450 nm was measured by microplate reader (Thermo, USA) to assess proliferative ability of cells.

### Flow cytometry analysis

About 1 × 10^6^ cells (apoptosis detection should include cells floating in culture supernatant) were obtained and washed by pre-chilled PBS. Cell apoptosis was analyzed by 5 μL Annexin V-FITC and 10 μL PI reagent (Multi Sciences, CHN), followed by 5 min incubation at 37 °C in a dark environment. Cell cycle was analyzed by 10 μL Permeabilization solution and 1 ml DNA Staining solution (Multi Sciences, CHN), followed by 30 min incubation at 37 °C away from light. Flow cytometry was used to analysis the cell apoptosis rate and cell cycle.

### Protein stability analysis

Protein stability was assessed by adding cycloheximide (CHX, MedChemExpress, CHN) at a concentration of 20 μg/ml to control and Y-27632 treated cells and culturing them at the specified time intervals. Western blotting was used to assess the degradation of β-catenin protein.

### Rat model experimental method

Part one. Animals and groups: twenty-four 6-week-old male Sprague–Dawley rats weighing between 200–250 g were randomly allocated into 3 groups, 8 rats in each group. (1) Control group (control), subcutaneous injection of corn oil (2) BPH rat group (T), subcutaneous injection of 2 mg/d testosterone propionate (3) Y-27632 treatment group, subcutaneous injection of testosterone (2 mg/d) alongside Y-27632 (dissolved in DMSO) injected directly to rat ventral prostate. Part two. Operation method: on the 14th day, operation was carried out under anesthesia with intraperitoneal administration of 35 mg/kg sodium pentobarbital. An incision was made in the middle of lower abdomen just above penis to help expose rat prostate. Intra-ventral prostatic injection (50 μL sterile saline as solvent) was performed with 30-gauge needle: treatment group was injected with 10 nmol Y-27632, alongside control group and T group were given DMSO with the comparable concentration. Following above injection, the incision was sutured under anesthesia with 2% lidocaine. Part three. Sample sampling: On the 28th day, euthanasia was performed via intraperitoneal administration of excess sodium pentobarbital (120 mg/kg), after which rat prostate, bladder and seminal vesicle were extracted for collection. A schematic illustration of the in vivo experimental procedure was shown in Fig. S6a. (Reagent source: corn oil and Y-27632 from MedChemExpress, CHN, testosterone propionate from Sigma-Aldrich, USA, sodium pentobarbital from Abbott Laboratories, USA). Animal experiments were approved by the Experimental Medical Ethics Committee of Zhongnan Hospital before commencing, and experiments were carried out in Animal Center of Zhongnan Hospital.

### Total RNA isolation, reverse transcription, and qRT-PCR analysis

Total RNA was isolated by RNA Isolation Reagent, and the concentration and quality were measured by NanoPhotometer spectrophotometer (Thermo, USA). The extracted RNA was reverse transcribed to synthesize cDNA with the Reverse Transcription Kit. qRT-PCR was conducted using the Universal SYBR Green qPCR Kit. The PCR program was as follows: 95 °C for 20 s; 39 cycles of 95 °C for 10 s, 60 °C for 30 s. Finally, gene expression levels were normalized to those of GAPDH, then analyzed using the 2^−ΔΔCt^ method. The reagents in this method were from Vazyme, CHN. All primer sequences were provided in Table. S4.

### Western blotting

Tissues and cells were subjected to ultrasonic treatment in RIPA Lysis buffer supplemented with phenylmethanesulfonyl fluoride and phosphatase inhibitors (Sigma-Aldrich, USA) for 30 min, then centrifuged in a pre-cooled centrifuge at 14,000 g for 10 min at 4 °C, the supernatant was retained. The protein lysate concentrations were quantified using the bicinchoninic acid assay. After separated by SDS–polyacrylamide gel electrophoresis (SDS-PAGE), the protein extracts were transferred onto a polyvinylidene fluoride (PVDF) membrane (Millipore, USA) with the wet transfer system (Bio-Rad, USA). Next, 5% skim milk was used to block the membrane at room temperature for 2 h. Appropriate primary antibodies (Table. S1) were applied and incubated overnight at 4 °C. Following multiple washes with TBST buffer, secondary antibodies (Table. S2) were incubated for 2 h at normal temperature. Immunoblots were detected using the Enhanced Chemiluminescence Kit (Thermo, USA) and analyzed by ImageJ software.

### Statistical analyses

Data were shown as the mean ± standard deviation (SD) from at least three independent experiments. GraphPadPrism8.0 software was used for statistical analysis. Group comparisons were made using either Student’s t-test or one-way ANOVA. A *p*-value less than 0.05 was used to indicate statistically significant. The corresponding significance levels were indicated by asterisks: * *p* < 0.05, ** *p* < 0.01, *** *p* < 0.001.

## Supplementary Information

Supplementary Material 1.

## Data Availability

The data used to support the results of the present study are available from the corresponding author upon reasonable request.

## Abbreviations

BPH: Benign prostatic hyperplasia
CCK-8: Cell counting kit-8
CHX: Cycloheximide
DBP: Di-n-butyl phthalate
EFS: Electric field stimulation
EMT: Epithelial-mesenchymal transition
FAs: Focal adhesions
FBS: Fetal bovine serum
H&E: Hematoxylin–eosin
IC50: Half-maximal inhibitory concentration
LEF: Lymphoid enhancer factor
LUTS: Lower urinary tract symptoms
MLC: Myosin light chain
PBS: Phosphate buffered saline
PE: Phenylephrine
PFA: Paraformaldehyde
PSA: Prostate-Specific Antigen
PVDF: Polyvinylidene fluoride
qRT-PCR: Quantitative real time PCR
RBD: Rho-binding domain
ROCK: Rho-associated protein kinase
SD: Standard deviation
SDS/PAGE: SDS–polyacrylamide gel electrophoresis
SiRNA: Small interfering RNA
T: Testosterone
TMA: Tissue microarray
