# Supplementary material for: Y-27632 targeting ROCK1&2 modulates cell growth, fibrosis and epithelial-mesenchymal transition in hyperplastic prostate by inhibiting β-catenin pathway
Source: Mol Biomed. 2024 Oct 26;5:52. doi: 10.1186/s43556-024-00216-9 (PMC11511810; doi:10.1186/s43556-024-00216-9)
Supplement: Supplementary file 1 — Supplementary Material 1. [file 43556_2024_216_MOESM1_ESM.pdf]

Supplementary figure 1

Immunohistochemistry analysis of TMA

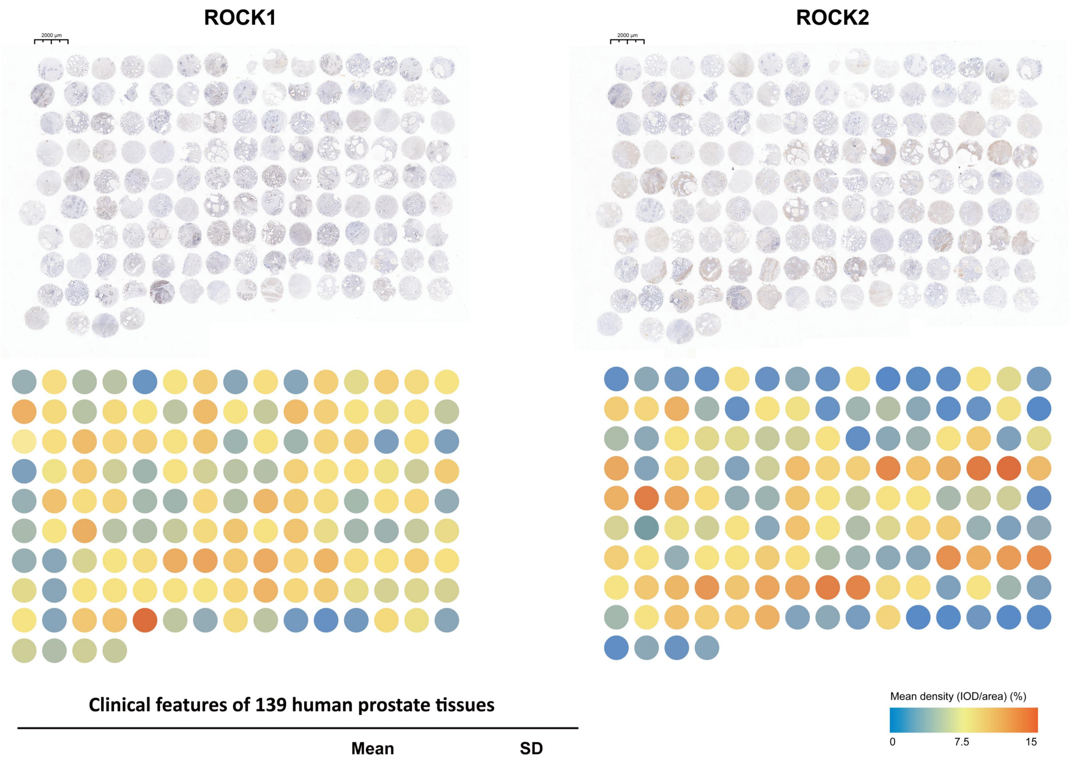

Clinical features of 139 human prostate tissues

	Mean	SD
Age (years)	67.2	12.4
BMI (body mass index) (kg/m²)	23.1	2.9
PV (prostate volume) (cm³)	52.5	34.7
N (nocturia)	4.4	1.7
fPSA (free prostate specific antigen) (ng/ml)	1.4	1.6
tPSA (total prostate specific antigen) (ng/ml)	6.2	8.2
fPSA/tPSA	0.3	0.1
Qmax (maximum flow rate) (ml/s)	11.0	8.9
RU (residual urine) (ml)	94.0	112.1

The correlation analysis of clinical parameters and ROCK in 139 human prostate tissues

	ROCK1		ROCK2	
	Pearson correlation	p-value	Pearson correlation	p-value
Age	0.04883	0.5695	0.2616	0.0019**
BMI (body mass index)	0.01915	0.8405	0.1645	0.0817
PV (prostate volume)	0.07434	0.4445	0.1348	0.1642
N (nocturia)	-0.008549	0.9599	-0.02248	0.8949
fPSA (free prostate specific antigen)	0.08929	0.3536	0.2637	0.0054**
tPSA (total prostate specific antigen)	0.1923	0.0432*	0.2773	0.0032**
fPSA/tPSA	-0.2239	0.0187*	-0.1565	0.1025
Qmax (maximum flow rate)	-0.1385	0.4820	-0.1187	0.5475
RU (residual urine)	-0.1584	0.3291	0.1459	0.3691

*. p<0.05 (2-tailed) **. p<0.01 (2-tailed)

Supplementary figure 1. Correlation analysis between ROCK immunohistochemical staining and clinical parameters in tissue microarray. Collection and treatment of all human specimens were in accordance with guidelines of the Ethics Committee at Zhongnan Hospital of Wuhan University. The 139 prostate tissue samples were obtained from young brain-dead donors undergoing organ donation or patients undergoing transurethral prostatectomy at the Zhongnan Hospital of Wuhan University. All specimens were stained with hematoxylin and eosin (H&E) staining and confirmed by a senior pathologist and representative fields were selected. * $p < 0.05$, ** $p < 0.01$. The scale bars are 2000 μm.

Supplementary figure 2

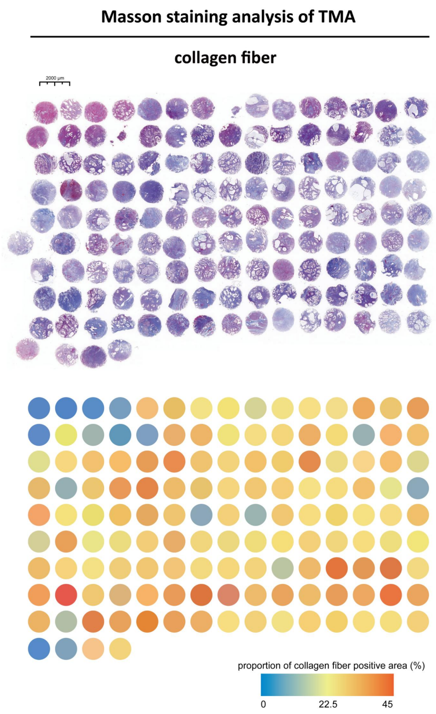

The correlation analysis of clinical parameters and the proportion of collagen fiber positive area in 139 human prostate tissues

	proportion of collagen fiber positive area	
	**Pearson correlation**	**p-value**
Age	0.2092	0.0138*
BMI (body mass index)	0.1585	0.0936
PV (prostate volume)	-0.02175	0.8232
N (nocturia)	-0.1054	0.5348
fPSA (free prostate specific antigen)	-0.01329	0.8904
tPSA (total prostate specific antigen)	-0.07757	0.4184
fPSA/tPSA	0.03139	0.7447
Qmax (maximum flow rate)	-0.006976	0.9719
RU (residual urine)	-0.2599	0.1053

*. p<0.05 (2-tailed)

Supplementary figure 2. Correlation analysis between Masson staining and clinical parameters in tissue microarray. prostate epithelial cells were stained orange, SM cells were stained red, and collagen fibers were stained blue, * $p<0.05$. The scale bars are 2000 μm.

Supplementary figure 3

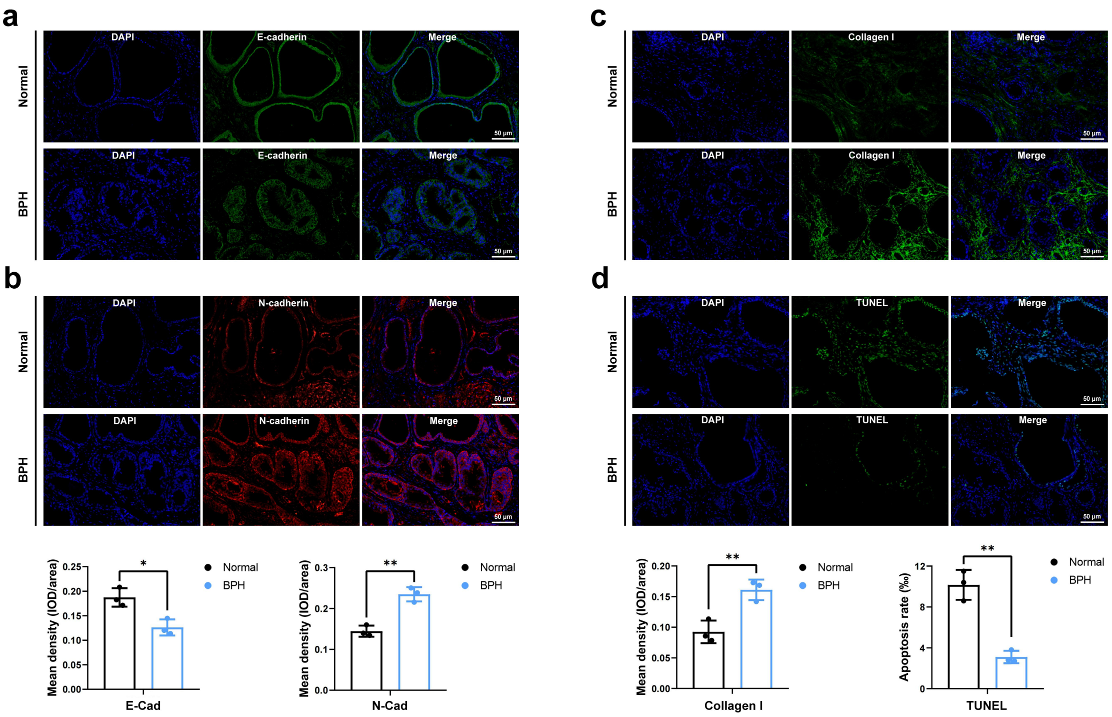

Supplementary figure 3. EMT, fibrosis and apoptosis related human prostate tissues staining. (**a**) Immunofluorescence staining of E-Cad for normal human prostate and BPH prostate. DAPI (blue) indicated nuclear staining and FITC-immunofluorescence (green) indicated E-Cad protein staining. (n=3). (**b**) Immunofluorescence staining of N-Cad for normal human prostate and BPH prostate. DAPI (blue) indicated nuclear staining and Cy3-immunofluorescence (red) indicated N-Cad protein staining. (n=3). (**c**) Immunofluorescence staining of Collagen I for normal human prostate and BPH prostate. DAPI (blue) indicated nuclear staining and FITC-immunofluorescence (green) indicated Collagen I protein staining. (n=3). (**d**) The TUNEL staining for normal human prostate and BPH prostate. DAPI (blue) and fluorescence-labeled images (green) were merged. (n=3). Data were expressed as mean ± SD. * $p < 0.05$, ** $p < 0.01$. The scale bars are 50 μm.

Supplementary figure 4

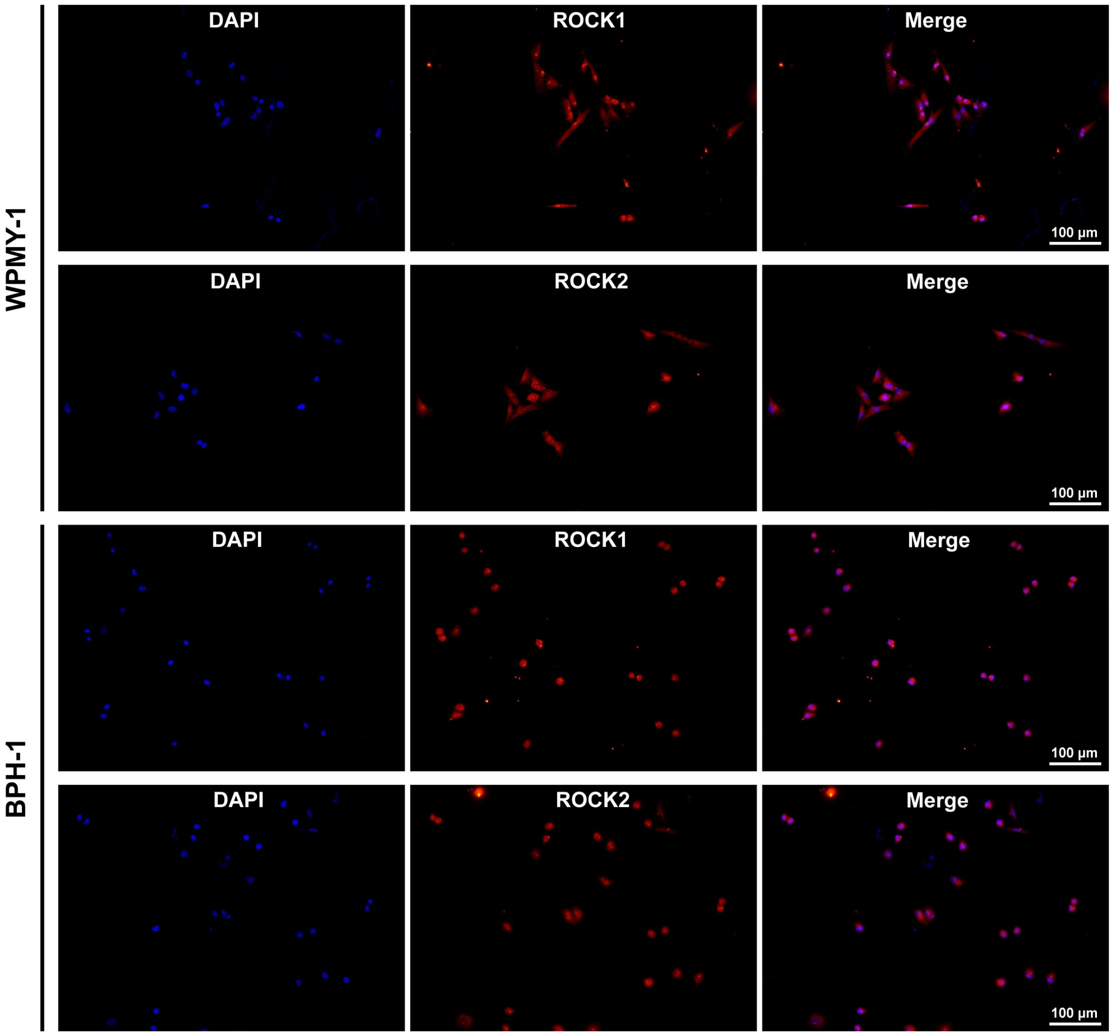

Supplementary figure 4. ROCK immunofluorescence staining of human prostate cell lines. Immunofluorescence staining of ROCK1 or ROCK2 for human prostate cell lines. DAPI (blue) indicated nuclear staining and Cy3-immunofluorescence (red) indicated ROCK1 or ROCK2 protein staining. The scale bars are 100 μm.

Supplementary figure 5

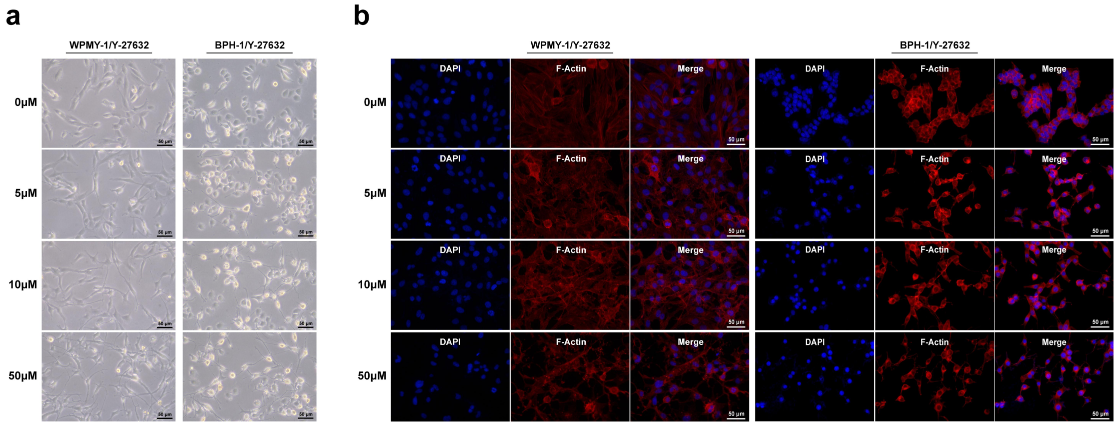

Supplementary figure 5. Cell morphology and cytoskeleton staining of human prostate cell lines. (**a**) Bright-field images of WPMY-1 and BPH-1 after Y-27632 treated. (**b**) F-Actin staining of WPMY-1 and BPH-1 after Y-27632 treated. The scale bars are 50 μm.

Supplementary figure 6

a

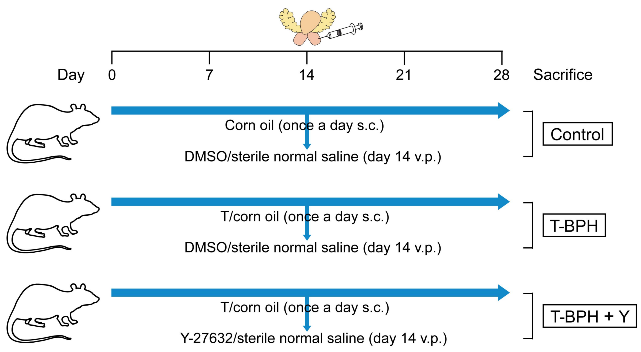

b

Variation of biometric and physiological parameters in Control, T and T+Y-27632 rats

Group	Body weight (g) Initial	Body weight (g) Final	Ventral prostate weight (mg)	Seminal vesicles weight (mg)	Prostate index	
Control	235.8(7.8)	455.8(35.1)	452.5(84.1)	890.0(126.3)	1.0(0.2)	
T	239.4(7.3)	399.1(32.0)**	1286.3(234.3)***	2087.5(329.1)***	3.3(0.8)***	(vs.Control)
T + Y-27632	237.6(10.4)	398.0(33.0)	921.3(243.6)**	2227.5(341.0)	2.3(0.5)**	(vs.T)

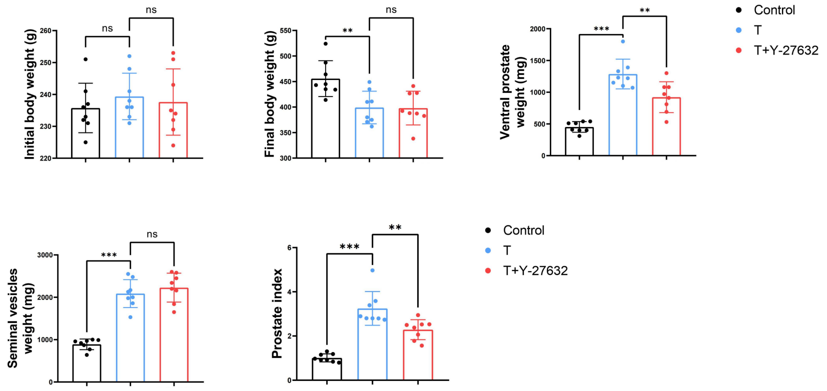

Supplementary figure 6. *In vivo* experimental methodology and the analysis of biological parameters. (**a**) The schematic of the *in vivo* experimental protocol in rats. (**b**) Variation of biometric and physiological parameters in Control, T and T+Y-27632 rats. (n=8). Data were expressed as mean ± SD. ns means no significant difference, ** $p < 0.01$, *** $p < 0.001$.

Supplementary Table S1: List of primary antibodies

Antigens	Species antibodies raised in	Dilution used	Supplier
ROCK1	Rabbit monoclonal	1:500(WB)	abcam
		1:100(IF)	Ab134181
		1:100(IHC)	
ROCK2	Rabbit monoclonal	1:10000(WB)	abcam
		1:100(IF)	Ab125025
		1:100(IHC)	
Ki-67	Rabbit polyclonal	1:1200 (IF)	Servicebio
			GB111141
Bcl-2	Mouse monoclonal	1:1000(WB)	Proteintech
			68103-1-Ig
BAX	Rabbit polyclonal	1:1000(WB)	Abclonal
			A12009
α-SMA	Mouse monoclonal	1:1000(WB)	Servicebio
		1:100(IF)	GB13044
Collagen I	Rabbit polyclonal	1:1000(WB)	Abclonal
		1:100(IF)	A1352
Cytokeratin 18	Rabbit monoclonal	1:100(IF)	Abclonal
			A19778
N-Cad	Mouse monoclonal	1:1000(WB)	Proteintech
			66219-1-Ig
E-Cad	Rabbit polyclonal	1:1000(WB)	Proteintech
			20874-1-AP
Vimentin	Rabbit monoclonal	1:1000(WB)	Abclonal
			A19607
β-catenin	Mouse monoclonal	1:5000(WB)	Proteintech
		1:200(IF)	66379-1-Ig
		1:250(IHC)	

C-MYC	Mouse monoclonal	1:1000(WB)	Proteintech<br>67447-1-Ig
Survivin	Rabbit polyclonal	1:1000(WB)	Affinity Biosciences<br>AF0617
Snail	Rabbit polyclonal	1:1000(WB)	Abclonal<br>A11794
p-Smad3	Rabbit polyclonal	1:1000(WB)	Abclonal<br>AP1263
p-Smad2	Rabbit polyclonal	1:1000(WB)	Abclonal<br>AP0925
TGF-β1	Rabbit polyclonal	1:1000(WB)	Abclonal<br>A2124
GAPDH	Rabbit polyclonal	1:10000(WB)	Abclonal<br>AC027

Supplementary Table S2: List of secondary antibodies

Secondary detection system used	Host	Dilution used	Supplier
HRP-conjugated Affinipure Goat Anti-Mouse IgG(H+L)	Goat	1:10000 (WB)	Proteintech SA00001-1
HRP-conjugated Affinipure Goat Anti-Rabbit IgG(H+L)	Goat	1:10000 (WB)	Proteintech SA00001-2
Anti-rabbit IgG (H+L), F(ab')2 fragment (Alexa Fluor® 488 Conjugate)	Goat	1:50 (IF)	Cell Signaling Technology, USA, cat. no. 4412
Anti-mouse IgG (H+L), F(ab')2 Fragment (Alexa Fluor® 488 Conjugate)	Goat	1:50 (IF)	Cell Signaling Technology, USA, cat. no. 4408
Anti-rabbit IgG (H+L), F(ab')2 Fragment (Alexa Fluor® 594 Conjugate)	Goat	1:50 (IF)	Cell Signaling Technology, USA, cat. no. 8889
Anti-mouse IgG (H+L), F(ab')2 Fragment (Alexa Fluor® 594 Conjugate)	Goat	1:50 (IF)	Cell Signaling Technology, USA, cat. no. 8890
Hoechst 33342 (1 mg/ml) nucleic acid staining (DAPI)	-	1:750 (IF)	Molecular Probes/Invitrogen, Carlsbad, CA, USA, cat. no. A11007

Supplementary Table S3: Sequences of each siRNA

siRNA	Sequence (5' to 3')	
H6093-SiROCK1-1	sense	GGAAGGUAUAUGCUAUGAA
	antisense	UUCAUAGCAUAUACCUUCC
H6093-SiROCK1-2	sense	GGUUGGAACUUACAGUAAA
	antisense	UUUACUGUAAGUUCCAAACC
H6093-SiROCK1-3	sense	AUCACCUACAAGCAUUUCG
	antisense	CGAAAUGCUUGUAGGUGAU
H9475-SiROCK2-1	sense	CCUUGUAAACCUUAUGAGU
	antisense	ACUCAUAAGGUUUACAAGG
H9475-SiROCK2-2	sense	GAACAUCUUAGCAAUGAGA
	antisense	UCUCAUUGCUAAGAUGUUC
H9475-SiROCK2-3	sense	GAAUAUGUGGCCUAGAAGA
	antisense	UCUUCUAGGCCACAUAUUC

Supplementary Table S4: Primer sequences used for qRT-PCR

Gene	Primer	Primer sequence (5' to 3')
ROCK1	Forward	AACATGCTGCTGGATAAATCTGG
	Reverse	TGTATCACATCGTACCATGCCT
ROCK2	Forward	TTGCTCTGGATGCAATACACTC
	Reverse	TCTCGCCCATAGAAACCATCA
GAPDH	Forward	GGAGCGAGATCCCTCCAAAAT
	Reverse	GGCTGTTGTCATACTTCTCATGG
